# Postoperative Glans Color Changes Following Penile Prosthesis Implantation: Not Always Glans Ischemia

**DOI:** 10.3390/jcm15031267

**Published:** 2026-02-05

**Authors:** Josep Torremadé Barreda, Maurizio D’Anna, Xavier Bonet Puntí, Juan Ignacio Martínez Salamanca, Antonio Alcaraz Asensio, Lluis Peri Cusí

**Affiliations:** 1 Hospital Clínic de Barcelona, 08036 Barcelona, Spain; 2 TAUS Clinics, Andrología Barcelona, 08021 Barcelona, Spain; 3 TAUS Clinics, Andrología Mallorca, 07010 Palma, Spain; 4 Lyx Instituto de Urología, 28003 Madrid, Spain

**Keywords:** penile prosthesis, glans ischemia, penile necrosis, erectile dysfunction surgery, postoperative complications

## Abstract

**Background/Objectives:** Penile prosthesis implantation is a safe and effective treatment for erectile dysfunction, with low complication rates. Glans ischemia is a rare but serious postoperative complication that can lead to irreversible tissue loss. However, not all postoperative glans color changes reflect true ischemia, and distinguishing reversible from irreversible perfusion compromise remains challenging. The objective was to describe the clinical course, management, and outcomes of four patients who developed glans color changes following penile prosthesis implantation, emphasizing the role of glans sensibility in guiding treatment. **Methods:** We conducted a retrospective case series supplemented with a narrative literature review. Clinical data were obtained from medical records, operative reports, and follow-up visits. Literature searches were performed using PubMed, Scopus, and Google Scholar. **Results:** Four patients developed postoperative glans discoloration. Two patients, with preserved glans sensibility and no evidence of tissue necrosis, were managed conservatively with cylinder deflation and removal of compressive dressings, resulting in full recovery without tissue loss. Two patients, who exhibited impaired glans sensitivity, developed progressive ischemia. One had prior pelvic radiation, and the other underwent combined grafting and glanspexia. Both required surgical debridement and reconstruction, with permanent tissue loss. **Conclusions:** Glans color changes after penile prosthesis implantation do not always indicate irreversible ischemia. Preserved glans sensibility is a useful clinical marker of potentially reversible perfusion compromise and may support a conservative management strategy with close monitoring. Conversely, loss of sensation and necrosis should prompt urgent consideration of prosthesis explantation to prevent further tissue loss.

## 1. Introduction

Penile prosthesis implantation has become a safe and reproducible procedure, with low complication rates and minimal morbidity. Since the introduction of antibiotic-soaked devices, infection rates are approximately 1% [1], and functional prosthesis survival has been reported at 85% at 10 years [2].

One of the most dreaded complications is glans ischemia, which can lead to irreversible tissue loss. Although rare, it is potentially devastating.

Due to underreporting, standardized management protocols are lacking. Traditionally, management is aimed at reducing external and internal compression of penile vascular flow. Most authors advocate for immediate implant removal, since leaving the device in place is thought to inevitably worsen ischemia and tissue loss [3]. Conversely, glans ischemia has also been described after circumcision, where restitutio ad integrum has occasionally been achieved with conservative measures [4].

Because of the very low incidence of post-prosthesis glans ischemia, comparative studies between immediate prosthesis removal and a conservative “wait-and-see” strategy are unlikely. Most of the available literature consists of case reports and small series from high-volume implant centers. Importantly, changes in glans coloration after penile prosthesis implantation do not necessarily reflect ischemia, even when they appear clinically suggestive.

We present a series of four patients who developed unusual glans color changes following penile prosthesis implantation, each with distinct outcomes and management strategies.

## 2. Materials and Methods

This study was designed as a retrospective case series from patients treated between 2016 and 2024, supplemented with a narrative literature review. Clinical data were obtained retrospectively from the patients’ medical records, operative reports, and follow-up visits. No experimental interventions were performed, and management followed standard clinical practice. Patients provided informed consent for the use of anonymized clinical data and images for publication. 

For the narrative literature review, PubMed/MEDLINE, Scopus, and Google Scholar were searched up to June 2025. Keywords included *penile prosthesis*, *penile implant*, *glans ischemia*, *penile necrosis*, and *ischemic complications*. Articles in English and Spanish were considered, and reference lists of relevant publications were screened for additional reports.

## 3. Case Presentation

Case 1: Favorable outcome with conservative treatment in a patient with a penile implant plus bovine pericardium ventral graft for Peyronie’s disease.

**Figure 1:**
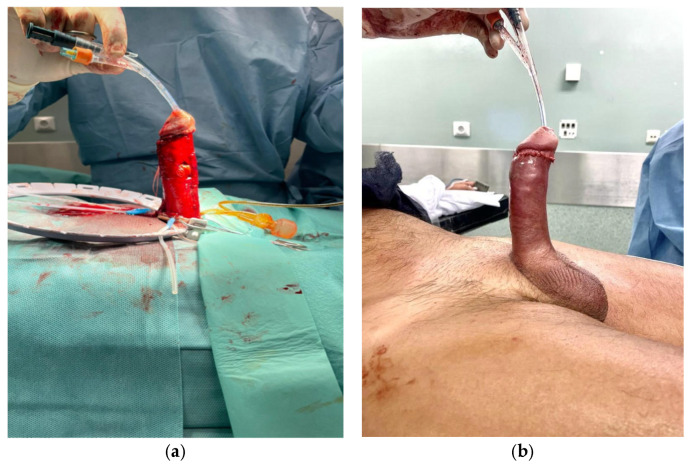
Case 1. Patient with a 90° ventral curvature and erectile dysfunction. (**a**) Ventral bovine pericardium grafting and penile implant, achieving penile straightening; (**b**) end result.

A 74-year-old man with a history of robotic-assisted radical prostatectomy, hypertension, and dyslipidemia presented with severe Peyronie’s disease, characterized by a 90° ventral curvature and erectile dysfunction. He underwent ventral bovine pericardium grafting combined with penile prosthesis implantation via a subcoronal approach. A Titan^®^ (Coloplast A/S, Humlebæk, Denmark) inflatable penile prosthesis was implanted, achieving complete penile straightening (Figure 1a). The prosthesis was inflated to approximately 70% capacity (Figure 1b). A closed-suction drain and compressive mummy-wrap dressing with a Peha-Haft^®^ (Paul Hartmann AG, Heidenheim, Germany) adhesive bandage were applied.

**Figure 2:**
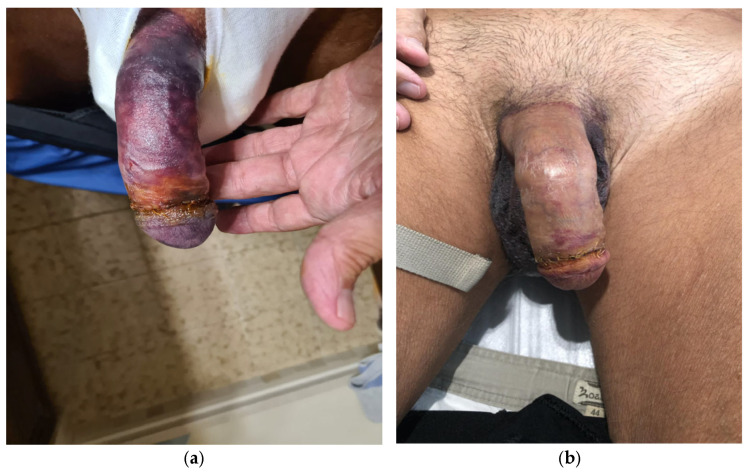
(**a**) First postoperative day. A dusky appearance of the glans and penile shaft, sensitivity to light touch was preserved, a conservative approach was chosen. (**b**) Seventh postoperative day. Ischemic changes have receded.

**Figure 3:**
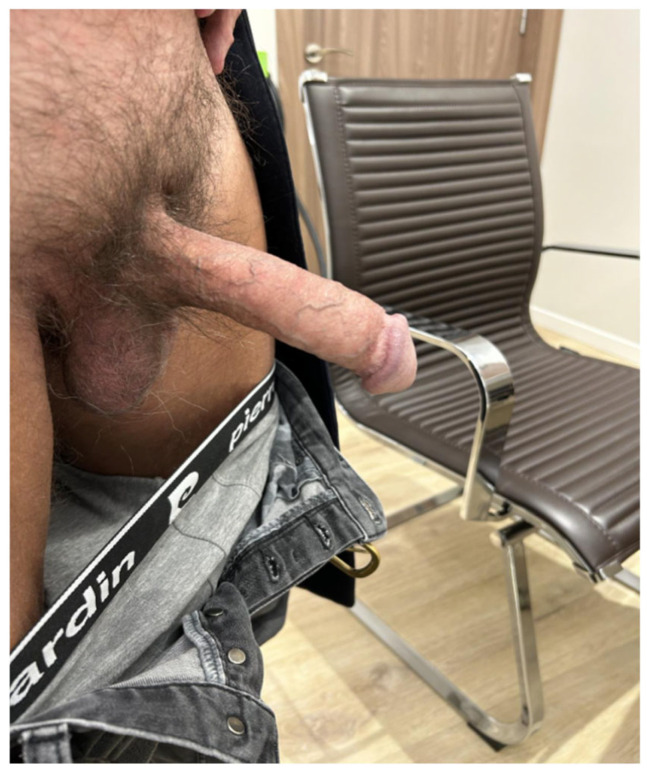
Outcome after 12 weeks. No prosthesis removal or surgical debridement was required.

On postoperative day 1, a dusky, darkened discoloration of the glans could be observed, suggestive of compromised perfusion. Notably, the dusky discoloration was not confined to the glans but involved the penile shaft as well (Figure 2a). The dressing was removed, and the prosthesis was fully deflated. Sensibility, assessed by light touch, remained intact. Management options were discussed with the patient, and a conservative observational approach was chosen. Over the following days, glans perfusion progressively improved, with near-complete normalization by postoperative day 7 (Figure 2b). The remainder of the postoperative course was uneventful. Prosthesis cycling was initiated at week 3, and the patient achieved satisfactory functional outcomes (Figure 3).

Case 2: Favorable outcome with conservative treatment after a penoscrotal penile implant.

A 52-year-old man with a history of type 2 diabetes mellitus and hypertension underwent penile prosthesis implantation via a penoscrotal approach. The procedure was uneventful. The cylinders were left inflated to approximately 70% capacity, a closed-suction drain was placed, and a compressive mummy-wrap dressing with adhesive gauze (Peha-Haft^®^) was applied.

**Figure 4:**
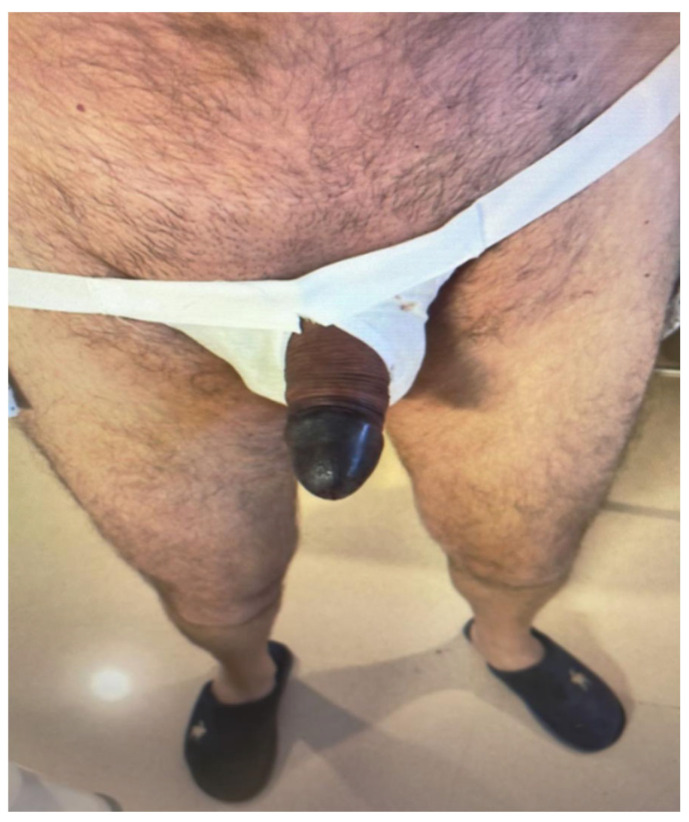
Case 2, first postoperative day. The compressive dressing was removed and the prosthesis fully deflated. Sensitivity was preserved.

On postoperative day 1, the glans appeared dusky. The dressing was immediately removed, and the prosthesis was fully deflated. The patient reported preserved glans sensitivity without pain or hypoesthesia, confirmed by light touch testing. Within hours, the glans developed a fully ischemic appearance (Figure 4). Sensitivity, reassessed with both light touch and pinprick, remained intact.

**Figure 5:**
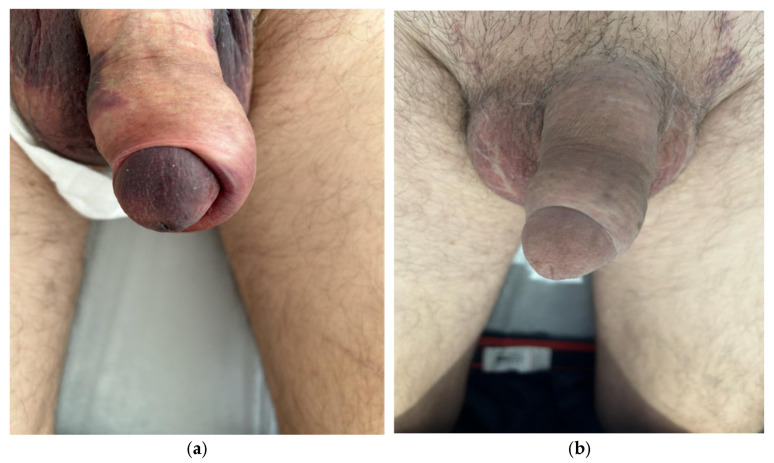
(**a**) Fifth postoperative day, an improved glans perfusion could be seen; (**b**) outcome after two weeks. Postoperative course was uneventful afterwards.

Management options—including urgent prosthesis removal versus conservative observation—were discussed with the patient, who opted for a conservative approach. Over the following days, glans perfusion progressively improved (Figure 5a), while sensitivity remained preserved. Prosthesis cycling was initiated at week 3, and penetrative intercourse was successfully resumed at 6 weeks. No debridement was required, and long-term outcomes were favorable (Figure 5b).

Case 3: Unfavorable outcome after a penile implant in a patient with a past medical history of radical prostatectomy and radiotherapy.

A 63-year-old man with a past medical history of chronic tobacco use, radical prostatectomy, and pelvic radiation underwent penile prosthesis implantation for erectile dysfunction. The procedure was performed via a transverse scrotal incision. The patient also presented with phimosis, and a circumcision was performed. The procedure itself was uneventful, and the cylinders were left partially inflated postoperatively. A compressive dressing and a closed-suction drain were applied as per routine practice.

**Figure 6:**
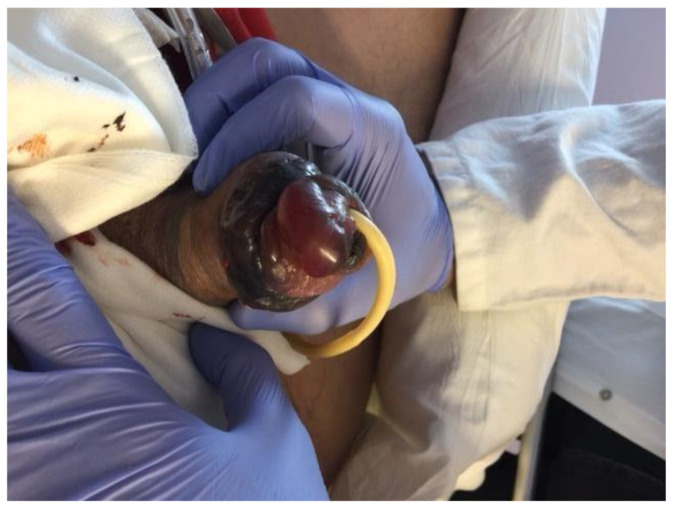
Case 3, first postoperative day. An ischemic glans with blistering and impaired sensitivity could be observed.

On postoperative day 1, the glans appeared ischemic, with blisters and a dusky appearance (Figure 6). The dressing was removed and the prosthesis deflated. Clinical assessment revealed impaired glans sensitivity, confirmed by light touch and pinprick testing. Despite conservative measures, glans perfusion did not improve.

**Figure 7:**
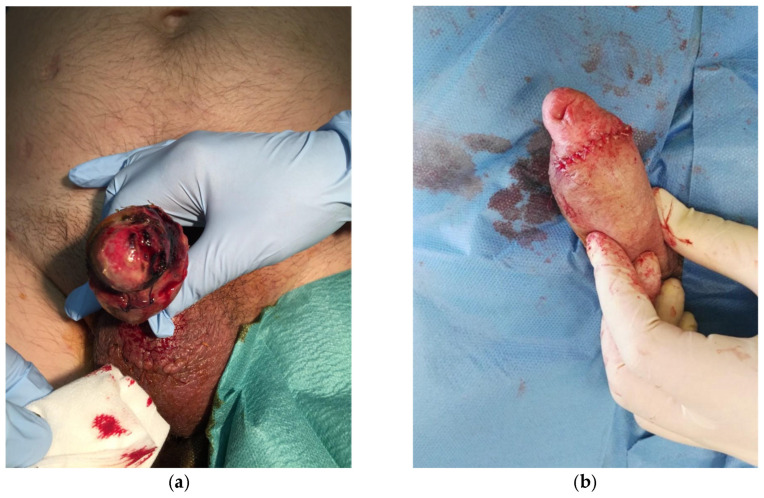
(**a**) Glans ischemia was established; prosthesis removal and penile debridement were required. (**b**) Result after prosthesis removal and penile debridement. Cosmetic outcomes were compromised.

The patient required multiple surgical debridements due to progressive tissue loss (Figure 7a). Sensibility remained impaired throughout the postoperative course. Ultimately, partial glans necrosis developed, with loss of glanular tissue. Long-term functional and cosmetic outcomes were significantly compromised (Figure 7b).

Case 4: Glans ischemia after a penile implant via a combined penoscrotal and subcoronal approach with grafting plus glanspexy.

A 52-year-old male with a history of diabetes mellitus, hypertension, and dyslipidemia was scheduled for penile prosthesis implantation due to Peyronie’s disease and erectile dysfunction. He underwent prosthesis placement combined with grafting and glanspexia, performed through scrotal and subcoronal incisions.

**Figure 8:**
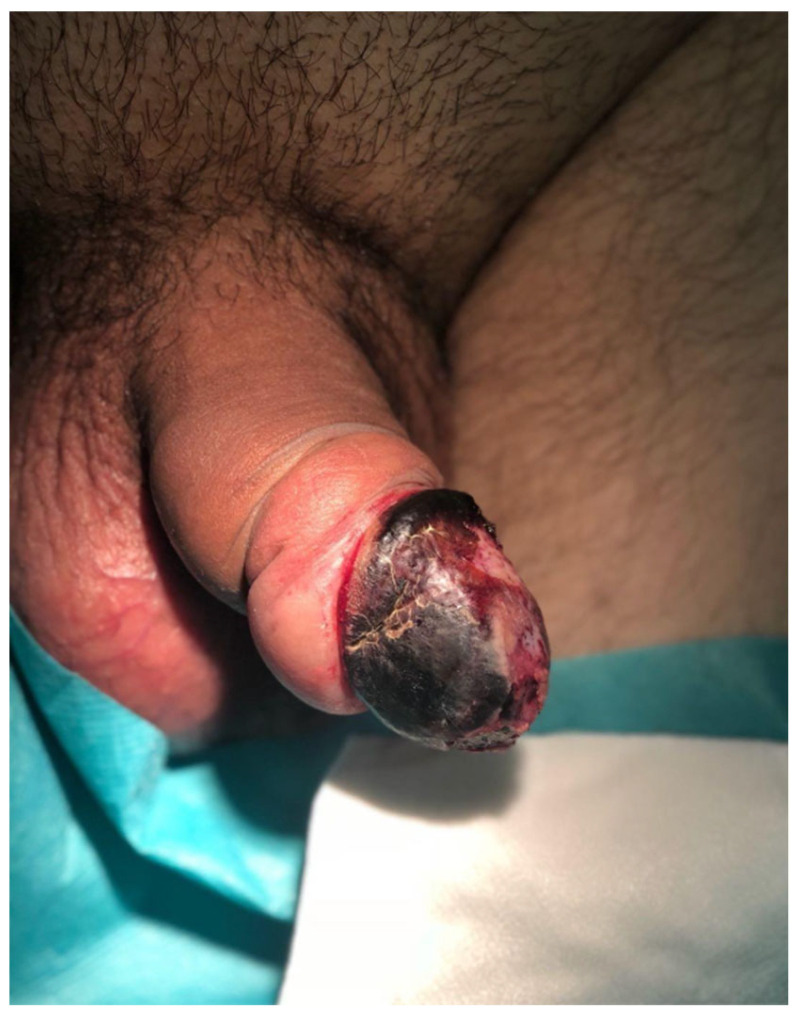
Case 4, first postoperative day. An ischemic glans with a dark eschar and impaired sensitivity prompted to urgent glans debridement.

**Figure 9:**
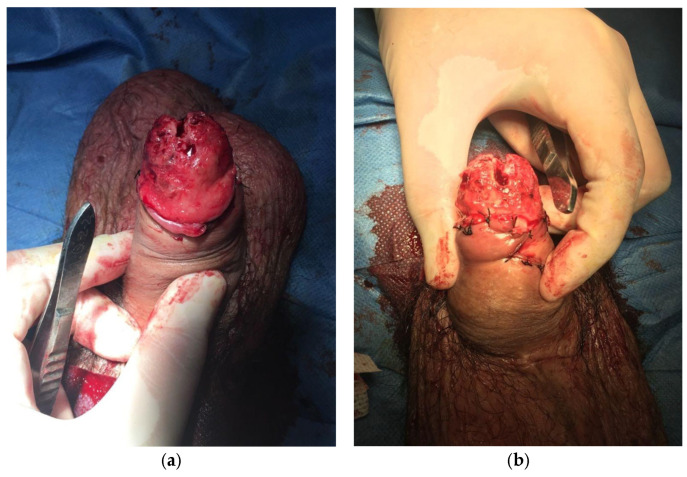
(**a**) Penile debridement, glans resurfacing was performed; (**b**) adjunctive ventral Z-plasty to reduce tension.

The immediate postoperative course was complicated by the development of glans ischemia, manifested as a glans covered by a dark eschar and with impaired sensibility (Figure 8). As necrosis was established, surgical debridement was required (Figure 9a). Reconstruction was performed with subsequent procedures to optimize functional and cosmetic outcomes (Figure 9b).

**Figure 10:**
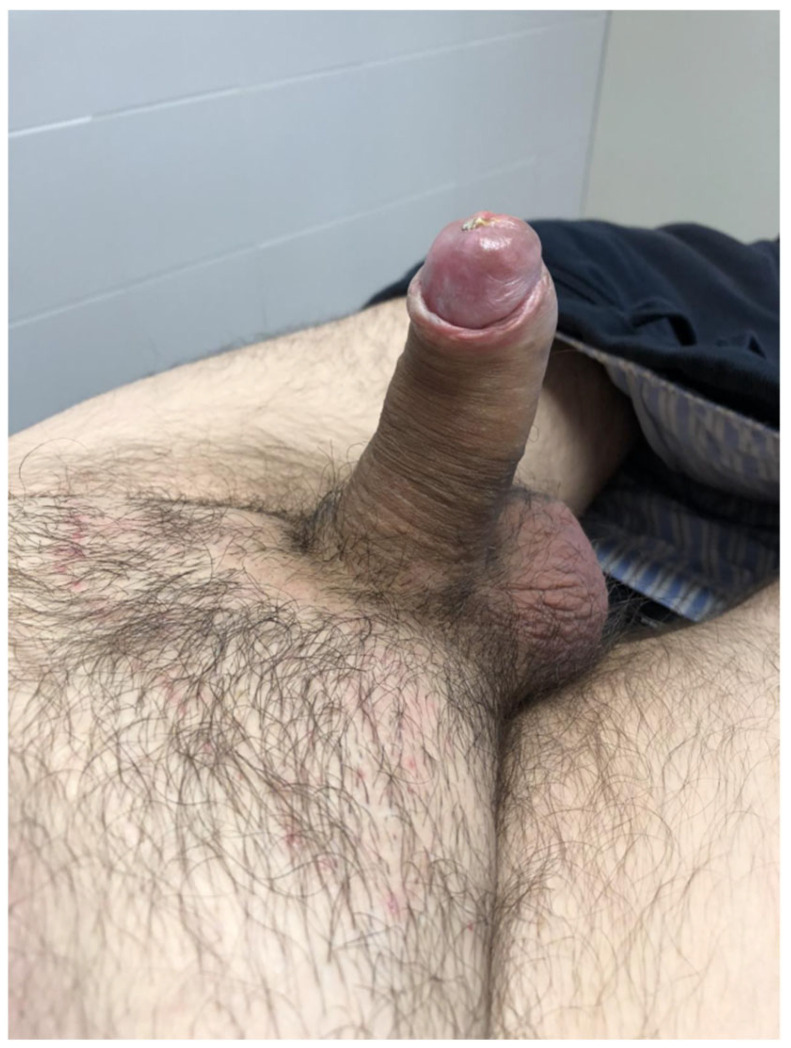
Result after debridement. There was some tissue loss, but overall, an acceptable cosmetic and functional result was achieved.

The patient’s recovery was prolonged but ultimately satisfactory, with stabilization of the penile tissue and partial recovery of function (Figure 10).

## 4. Discussion

Glans ischemia is a rare but potentially devastating complication of penile prosthesis implantation. Its true incidence remains uncertain due to underreporting. Large, prospective multicenter registries may clarify its frequency [5].

Prompt recognition and appropriate intervention are critical to prevent irreversible damage. The traditional management approach is urgent prosthesis removal to relieve intracorporeal pressure [6]. However, in some cases, ischemia may be transient and reversible with conservative measures, such as cylinder deflation and removal of external compression. In Table 1, we summarize the proposed indications for a conservative approach versus an urgent penile explant.

Vascular Considerations

Glans perfusion is supplied by branches of the internal pudendal artery, which divides into the penile and perineal arteries. The penile artery further branches into the cavernosal, dorsal penile, and bulbospongiosal arteries [3]. Extrinsic compression (tight dressings and subcoronal hematoma) and intrinsic compression (Foley catheter and inflated cylinders) may compromise this blood flow in susceptible patients.

Clinical Assessment of Ischemia

**Figure 11:**
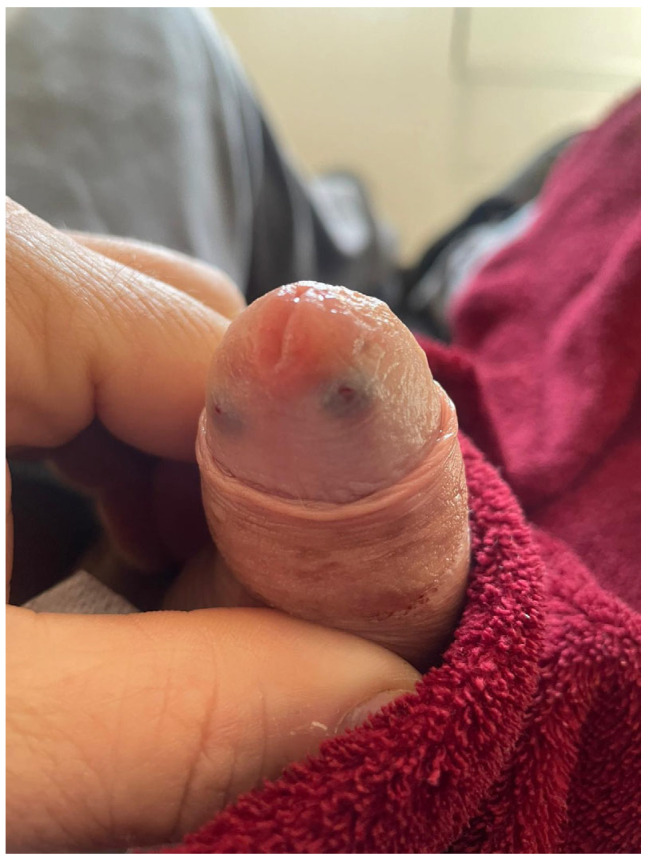
Small submucosal hematoma at the exit site of Keith needles.

**Figure 12:**
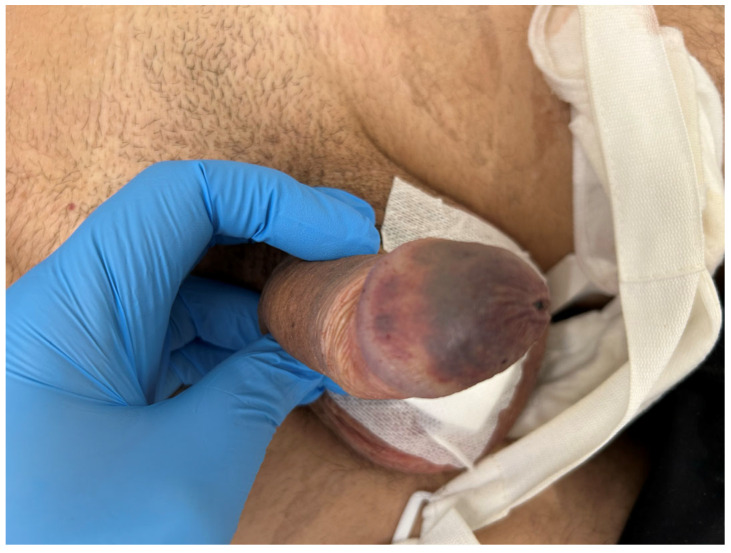
Diffuse submucosal hematoma, rarely comprising the whole glans, not suggestive of ischemia.

**Figure 13:**
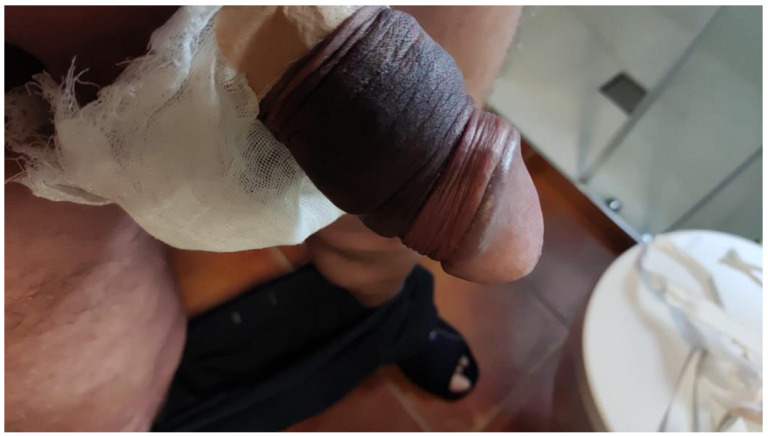
Ecchymosis is limited to the penile shaft, with no alterations in the glans.

- Inspection: A normal glans is pink or purplish. Small dorsal ecchymoses after prosthesis placement are common at Keith needle exit sites (Figure 11), which sometimes can be more diffuse but rarely affect the glans completely (Figure 12). In contrast, an ischemic glans appears diffusely pale, dusky, or black. In some patients, penile shaft and scrotal ecchymosis can be identified, with no glans involvement (Figure 13).
- Capillary refill and temperature: Delayed refill and coolness indicate poor perfusion.
- Palpation: A soft, pale, cool glans suggests arterial insufficiency, while a firm, congested glans may reflect venous outflow obstruction.
- Sensibility testing: Dorsal penile nerves (pudendal branches) should be tested using a light touch, pinprick, and temperature. Sensation must be compared to the proximal shaft. Preserved sensibility may indicate reversible ischemia. Loss of sensation suggests advanced vascular compromise.
- Pain: Disproportionate pain or new-onset numbness/tingling are early warning signs.
- Photography: Serial images aid monitoring.

Proposed Indications for Urgent Explantation

Patients with a dusky or necrotic glans with loss of light touch or pain sensation, a cold glans with absent capillary refill, progressive numbness, or anesthesia, are at higher risk of developing irreversible ischemia, with the possibility of tissue loss. Blistering can also mean that irreversible ischemia is present. In these cases, urgent prosthesis removal is warranted, diminishing intrinsic pressure.

Proposed Indications for a Conservative Approach

In patients with preserved glans sensibility, absence of tissue necrosis, or blistering, a conservative approach may be initially indicated. It is important to ensure close clinical follow-up. In such cases, extrinsic compression should be relieved and cylinders fully deflated. Photographic monitoring can help show the evolution of glans ischemia. The possibility of progression to irreversible ischemia must be disclosed to the patient.

## 5. Evidence in the Literature

Wilson et al. [6] reported 21 cases of glans ischemia following penile prosthesis implantation. Four patients (19%) were managed with prompt explantation and achieved complete recovery without tissue loss. In contrast, 17 patients (81%) were initially managed conservatively, but all ultimately required glans debridement or amputation. Based on these findings, the authors concluded that immediate explantation should be considered in high-risk patients. Notably, 86% of the affected individuals in this series had undergone a subcoronal incision or synchronous circumcision, suggesting a potential role of surgical approach in ischemic complications. 

Subsequently, Park et al. [7] analyzed 898 patients who underwent penile prosthesis implantation via a subcoronal approach and reported no cases of glans necrosis, highlighting the rarity of this complication and the difficulty in establishing clear risk factors. Other isolated reports have implicated prior pelvic surgery, radiation, diabetes mellitus, and vascular disease as potential contributors to compromised penile perfusion [8,9,10,11,12].

Our case series adds to this limited body of literature by underscoring the importance of glans sensibility as a key predictor of reversibility. In two of our cases, glans ischemia occurred, but sensitivity to light touch and pinprick was preserved, and both patients recovered fully under conservative management without tissue loss. In contrast, in the third and fourth cases, ischemia was accompanied by sensory impairment, and the patient developed progressive necrosis requiring repeated debridement with permanent tissue loss. 

Taken together, these findings suggest that preserved penile sensitivity may represent a clinically useful marker for safe conservative management, whereas loss of sensibility should raise concern for irreversible ischemia and prompt consideration of early prosthesis removal. Further prospective studies with larger cohorts are warranted to validate these observations and refine management algorithms (Table 2).

## 6. Study Limitations

This study has several limitations that should be acknowledged. First, the retrospective case series design limits causal inference and is subject to selection and reporting bias. The very small sample size and the absence of a denominator preclude any statistical analysis or estimation of incidence. As a result, the observations should be considered descriptive and hypothesis-generating rather than confirmatory.

Second, clinical assessment of glans ischemia and sensibility was not standardized or objectively quantified. Sensory evaluation relied on bedside testing with a light touch and pinprick, which may be subject to interobserver variability and patient-related factors such as anxiety or altered pain perception. Potential confounding variables, such as previous penile vascular parameters, the duration of the surgery, and the duration and type of compressive dressing, were not systematically controlled or analyzed. These factors may independently influence penile vascular perfusion and postoperative outcomes.

Finally, management decisions were individualized and based on shared decision-making rather than a predefined protocol, introducing treatment heterogeneity. While this reflects real-world practice, it limits reproducibility and generalizability. Larger, prospective, multicenter registries with standardized definitions, objective perfusion and sensibility assessments, and predefined management algorithms are needed to validate the proposed role of glans sensibility as a prognostic marker and to refine evidence-based recommendations for the management of postoperative glans color changes following penile prosthesis implantation.

## 7. Conclusions

Glans ischemia represents one of the most severe and feared complications of penile prosthesis surgery. Traditional management has emphasized early device explantation to prevent irreversible tissue loss. However, our case series suggests that in selected patients—particularly those with preserved glans sensibility and no evidence of necrosis—a carefully monitored conservative approach can lead to favorable outcomes and device preservation. Conversely, impaired sensibility should be regarded as a warning sign of irreversible ischemia, warranting urgent consideration of prosthesis removal.

Given the rarity of this complication, prospective data from multicenter registries are needed to better define risk factors, refine patient selection, and establish evidence-based management guidelines.

## Figures and Tables

**Table 1:** Proposed indications for a conservative approach or urgent penile implant removal.

Conservative Approach	Urgent Explantation
Preserved glans sensitivity	Dusky or necrotic glans with loss of light touch or pain sensation
Absence of tissue necrosis or blistering	Cold glans with absent capillary refill
Ability to ensure close clinical follow-up	Progressive numbness or anesthesia
	Blistering or necrosis

**Table 2:** Previous studies addressing glans ischemia after a penile implant.

Study	Year	Study Design	Patients	Type of Prosthesis	Management	Comments
Kadar et al. [9]	1995	Case report	1	Malleable	Conservative treatment	
Yildirim et al. [10]	2008	Case report	1	Malleable	Prosthesis removal + distal penectomy at postop week 4	
García-Gómez et al. [11]	2013	Case report	1	Inflatable	Glans necrosectomy at postop day 22.	Subcoronal incision + multiple incisions in the albuginea
Wilson et al. [6]	2017	Retrospective cases series	21	12 (57%) inflatable; 9 (43%) malleable.	4 patients (19%) required prosthesis removal; 17 (81%) conservative treatment, surgical debridement of glans necrosis	18 patients (86%) had a subcoronal incision (either for penile degloving or circumcision)
Hebert et al. [3,8]	2020	Case report	1	Inflatable	Prosthesis removal	Penoscrotal approach
Garrido-Abad P et al. [12]	2020	Case report	1	Inflatable	Pentoxifylline and hyperbaric treatment	Infrapubic approach

## Data Availability

The original contributions presented in this study are included in the article. Further inquiries can be directed to the corresponding author.
